# Drug-Induced Deaths in Türkiye, 2010–2024: Trends, Demographic and Substance Characteristics, and Comparison with Europe

**DOI:** 10.3390/jcm15218517

**Published:** 2026-11-02

**Authors:** Ömer Asan, Hüseyin Çağrı Şahin

**Affiliations:** Council of Forensic Medicine, Ministry of Justice, Istanbul 34196, Türkiye; hcagrisahin@gmail.com

**Keywords:** drug-induced deaths, synthetic cannabinoids, methamphetamine, new psychoactive substances, Türkiye, overdose mortality, drug policy

## Abstract

**Background/Objectives**: Türkiye is the most populous country reporting to the European drug monitoring system, yet its drug-induced deaths have rarely been studied in the international literature. We describe drug-induced mortality in Türkiye over fifteen years and compare its level and composition with those of European countries. **Methods**: We assembled all of the published data of the Council of Forensic Medicine, the national reference institution, on direct drug-induced deaths for 2010–2024, drawing on the national reports and European reporting channels, which proved to carry identical counts. Temporal trends were analysed using segmented Poisson regression. Rates were compared with a fixed panel of ten European Union (EU) countries with direct sex- and age-standardisation. **Results**: A total of 6129 deaths were recorded. Annual deaths rose seven-fold to 941 in 2017, fell by almost two thirds within two years, reached 246 in 2022 and rose again to 427 in 2024. The net rise in 2024 was confined to deaths recorded as involving synthetic cannabinoids. The wave was concentrated among men aged 15–44. At its height, mortality among young men exceeded the European panel by 1.6 to 2.8 times, while the all-ages rate remained below the European level. Women comprised 5.0% of decedents. Opioid involvement fell from 76.5% to 15.5%. Among single-substance deaths, the leading substance changed three times, from heroin to synthetic cannabinoids, MDMA, methamphetamine and back to synthetic cannabinoids. **Conclusions**: Responses built around opioids reach a minority of deaths. Türkiye needs monitoring that distinguishes changes in mortality from changes in substance attribution, and prevention directed at synthetic cannabinoids, stimulants and young men.

## 1. Introduction

Türkiye borders the European Union (EU), with a population of 85 million, yet little of what is known about its drug-induced deaths has reached the international literature. It is the most populous country outside the EU to report under the European drug-related deaths protocol, and it does so from a setting that differs from most of Europe in geography, demography and policy. It lies on the main route by which Afghan heroin reaches Western Europe, and in 2013 it seized more than twice the quantity of heroin seized by all EU member states together [1]. Its population is among the youngest in Europe, with a median age of 34 years [2]. Its drug policy is led by law enforcement, and drug use is a criminal offence [3]. European reports have repeatedly singled out its mortality data. Türkiye and the United Kingdom together accounted for three quarters of the deaths involving new psychoactive substances reported in Europe in 2016 and 2017 [4], and the EU drug agency has described the substance profile and the age profile of decedents in Türkiye as distinct from the general European pattern [5]. These observations rest on single years or single substances. No study has examined drug-induced deaths in Türkiye as a whole or over time.

The question matters beyond Türkiye because the overdose response developed in Europe and North America is built around opioids. Its two best-evidenced interventions, take-home naloxone and opioid agonist treatment, act on opioid overdose specifically [6,7]. The European Union Drugs Agency (EUDA) has organised its mortality monitoring and its guidance to services around an ageing population of opioid users, whose deaths increasingly involve methadone, benzodiazepines and potent synthetic opioids [8]. These interventions have no counterpart for deaths caused by synthetic cannabinoids or stimulants, for which no reversal agent and no agonist treatment exist [9,10]. In North America, where stimulants are involved in a growing share of overdose deaths, the limits of an opioid-centred response are already under debate [11].

Deaths involving new psychoactive substances also raise a measurement problem. Synthetic cannabinoids turn over rapidly, and their post-mortem detection depends on whether the compound in circulation is covered by the reference standards and analytical methods of the laboratory concerned [12,13,14]. Recorded synthetic cannabinoid involvement may therefore reflect analytical capacity as well as mortality. Any account of a national data must therefore distinguish changes in what occurred from changes in what could be detected.

Several features of the Turkish drug market and policy environment are relevant to the interpretation of its mortality data. The market has changed rapidly. Synthetic cannabinoids appeared in 2010 under the street name Bonzai and were soon detected in nearly all of the herbal mixtures submitted for forensic analysis in Istanbul [15]. Between 2011 and 2014, 125 compounds were brought under control and recorded synthetic cannabinoid incidents increased 71-fold [1]. Emergency departments in Istanbul recorded a surge of synthetic cannabinoid intoxications in the summer of 2014 [16]. Methamphetamine was first detected in 2009, and by 2017 it had brought more people into treatment in Türkiye than in any European country except Czechia and Slovakia [17]. In 2017 Türkiye recorded the second-largest quantity of 3,4-methylenedioxymethamphetamine (MDMA) seized in the world [18]. The policy response has followed a consistent line. National policy has relied on supply reduction and deterrence. Public alarm over synthetic cannabinoid deaths in 2014 prompted harsher penalties, including a 50% sentence increase for offences involving synthetic cannabinoids and the criminalisation of use [3]. Opioid agonist treatment has been available since 2010 and is based on buprenorphine–naloxone [19].

Despite the number of deaths recorded in Türkiye, these data have entered the international literature only twice, each time for a single substance class and a period ending in 2017. Roxburgh et al. [18] analysed MDMA-related deaths between 2009 and 2017, and López-Pelayo et al. [4] counted deaths involving new psychoactive substances in 2016 and 2017. The national drug reports published annual totals without breakdown until the 2018 edition. No population-based analysis has described the course of drug-induced mortality in Türkiye across time, substances, sex and age or compared its mortality rate and profile with those of European countries on a consistent basis.

We assembled all published national data on direct drug-induced deaths in Türkiye for 2010–2024, all of which originate from the CFM, drawing on the national reports and on the data that Türkiye reports to the European agency, and combined them with European source tables. We had four aims. The first was to describe the national data and identify its change points. The second was to characterise the evolution of the sex, age and substance profile of decedents. The third was to establish which substances and which age groups accounted for the principal changes. The fourth was to compare Türkiye with European countries on both the rate of drug-induced death and the demographic and substance profile of decedents.

## 2. Methods

### 2.1. Setting and Data Source

Forensic expert services in Türkiye are centralised in the Council of Forensic Medicine (CFM), the statutory expert body of the courts under the Ministry of Justice, with units in all 81 provinces. Deaths that are sudden, violent or otherwise suspicious are referred by the public prosecutor for external examination and, where needed, autopsy. The CFM is the national reference laboratory for forensic toxicology, and no other body in Türkiye produces population-level data on drug-induced deaths.

A direct drug-induced death is defined by the CFM according to the European Monitoring Centre for Drugs and Drug Addiction (EMCDDA) drug-related deaths protocol, Selection D [20]: a death occurring shortly after the consumption of one or more illicit substances, whether accidental, intentional or undetermined. The referral pathway, the case definition and the CFM’s single-producer role remained unchanged throughout the study period. Cases are classified after joint assessment of the investigation file, the crime scene report and a complete autopsy with toxicological analysis. The analytical panel for new psychoactive substances changed over the period (Supplementary Methods). No systematic record of the panels’ coverage, instrumentation or detection limits by year has been published. Deaths judged to result from one substance are recorded as single-substance deaths, and all others, including drug–alcohol combinations, as polysubstance deaths; both are attributions made by the CFM and are used here as published. Indirect drug-related deaths are recorded separately and were not included.

The CFM transmits its annual data to the Turkish Monitoring Centre for Drugs and Drug Addiction (TUBİM), the national focal point of the Reitox network within the Turkish National Police, which publishes them in the annual Turkish Drug Report [21] and reports them to the EMCDDA/EUDA. All mortality data used in this study were produced by the CFM, so the national reports, EMCDDA publications and EUDA source tables carry the same data through different channels; their consistency is documented in the Supplementary Methods.

### 2.2. Design, Variables and Measures

**Figure 1:**
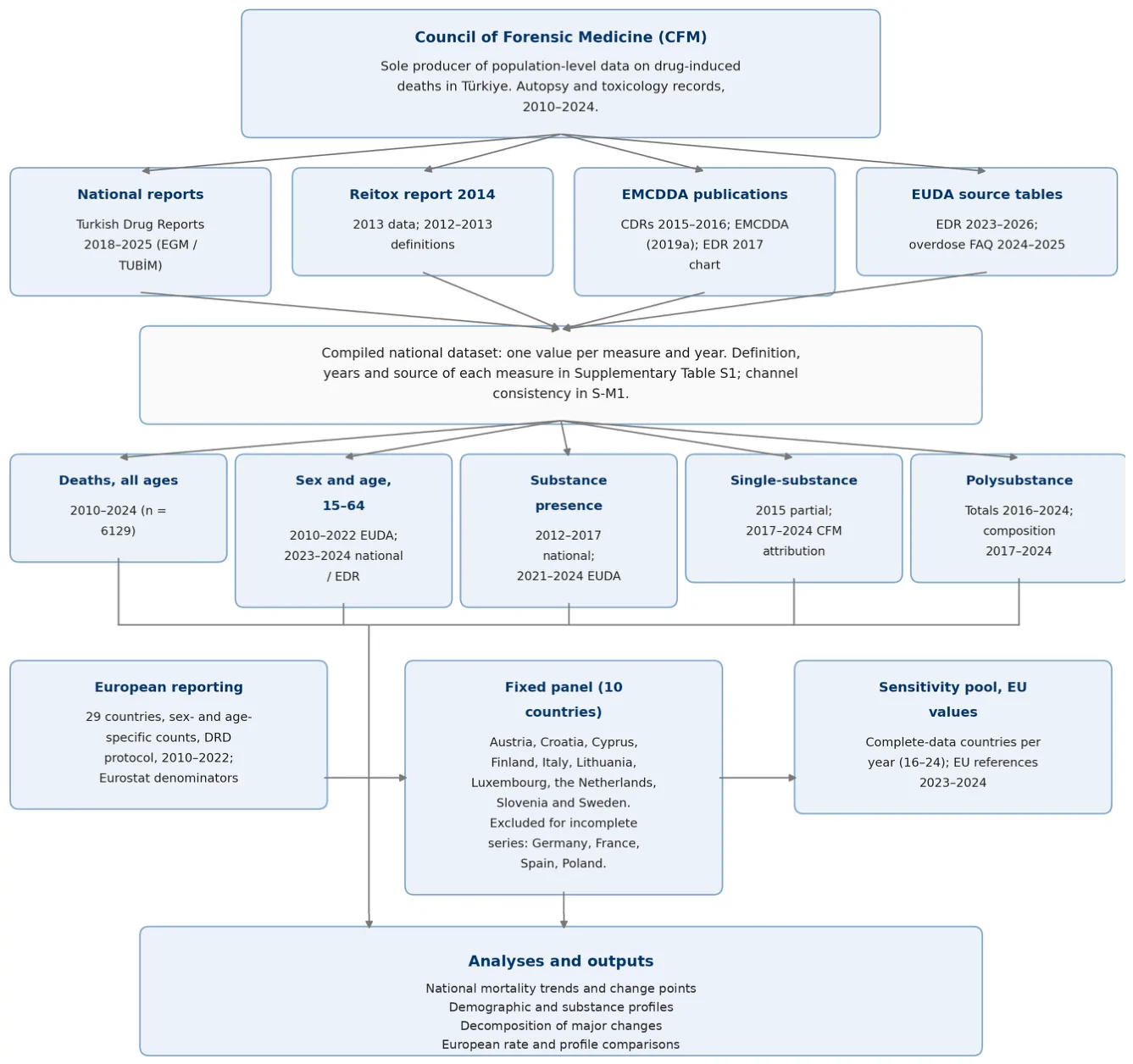
Data sources, measures and comparison sets. Flow of data from the Council of Forensic Medicine through four publication channels to the compiled dataset, the measures analysed with their years of availability, and the European comparison sets. Details of each measure are given in Supplementary Table S1; channel consistency is documented in S-M1.

The study is a retrospective analysis of routinely collected national mortality data for 2010–2024 by calendar year, reported in accordance with the REporting of studies Conducted using Observational Routinely-collected health Data (RECORD) statement [22]; all associations are ecological. Variables were annual deaths, sex, mean age, age distribution in the EUDA bands, substance presence (non-exclusive) for synthetic cannabinoids, methamphetamine, heroin, MDMA and any opioid, the CFM’s single-substance classification with the complementary polysubstance total, and substance involvement within polysubstance deaths. Substance presence is analytical detection at post-mortem examination and does not by itself establish an individual causal contribution to death, particularly where several substances are present; causal attribution exists in these data only in the single-substance classification, in which the CFM judges one substance to be solely responsible. Percentages were recalculated from published counts. Definitions, years of availability and source publications for each measure are given in Supplementary Table S1, and the flow of data from publication channels to the measures analysed and the European comparison sets is summarised in Figure 1. Three measurement differences matter for interpretation: opioid involvement is opiates present in the CFM’s tables (2012–2017) and any opioid present in the EUDA tables (2021–2024); age shares refer to ages 15–64 up to 2022 and to all deaths thereafter, a negligible difference; and the national polysubstance definition includes alcohol (Supplementary Methods). Trend comparisons were accordingly made within definition periods, and no trend statistic spans a change of definition. The only bridge across periods is the descriptive contrast of endpoints, which is conservative in direction for opioid involvement, since the earlier measure (opiates present) is narrower than the later one (any opioid present).

### 2.3. European Comparison

Reference values for 2024 at EU and country level were taken from the EUDA source tables of the European Drug Report 2026, and country rates for 2023 from the 2025 EUDA overdose FAQ [23]. Year-by-year comparison used the sex- and age-specific counts and Eurostat populations reported by all countries under the drug-related deaths protocol for 2010–2022. A fixed comparator panel comprised the ten EU countries with complete counts for both sexes and all three adult age bands in every year (Supplementary Table S3); Germany, France, Spain and Poland have incomplete series and were excluded. The ten-country criterion is therefore purely data-driven, requiring complete sex- and age-specific counts in every year of the period. Pooled panel rates are the sum of deaths over the sum of the population. The panel is a constant-composition comparator, not an estimate for the EU; as a sensitivity, the comparison was repeated with all countries with complete data in each year (16–24 countries). The observed-to-expected ratio applies the EU rate for 2024 to the Turkish population aged 15–64, with an exact Poisson interval treating the EU rate as fixed. The age-standardised rate ratio was computed by the direct method. Türkiye’s sex- and age-band-specific rates were applied to the panel’s sex- and age-specific population of the same year as the external standard, and the resulting standardised rate was divided by the pooled panel rate. This removes the distortion that Türkiye’s younger population structure introduces into crude-rate comparisons; crude and standardised ratios are reported side by side in the Results.

### 2.4. Statistical Analysis

Rates are given per million with exact Poisson 95% confidence intervals. The national series was described with a change-point Poisson regression of deaths on year with a log-population offset; among all configurations of zero to three change points at least two years apart, the one with the lowest Bayesian information criterion (2017, 2019, 2022) was retained. Segment slopes were estimated with quasi-Poisson variance and dispersion-scaled standard errors and expressed as annual percent changes. Because the change points were selected from the data, the sensitivity of the results to alternative numbers and placements of change points, together with a model with fixed change points at 2017 and 2022, is reported as a sensitivity analysis (Supplementary Methods), and inference is conditional on the selected configuration. Trends in proportions (female share, age shares and polysubstance share, over the periods reported) were estimated with quasi-binomial logistic regression and expressed as odds ratios per year; the trend in mean age after 2019 was estimated by ordinary least squares. Change in the composition of single-substance deaths over 2019–2024 was tested with the chi-square test using four categories (synthetic cannabinoids, methamphetamine, MDMA, and all other substances combined). Changes from 2023 to 2024 were expressed as rate ratios with log-normal confidence intervals and exact conditional tests; overlapping groups were compared descriptively. The Turkish-to-panel rate ratio was analysed on the log scale with segmented regression at the 2017 and 2019 change points and Newey–West standard errors (lag 1). Tests were two-sided without adjustment for multiplicity; the analysis is descriptive and exploratory, no single confirmatory hypothesis was pre-specified, and *p*-values are reported as descriptive measures of evidence rather than as confirmatory tests. Analyses used Python 3.12 (statsmodels 0.14.5 and SciPy 1.16.3).

## 3. Results

### 3.1. Temporal Pattern and Population-Based Mortality Rates

**Figure 2:**
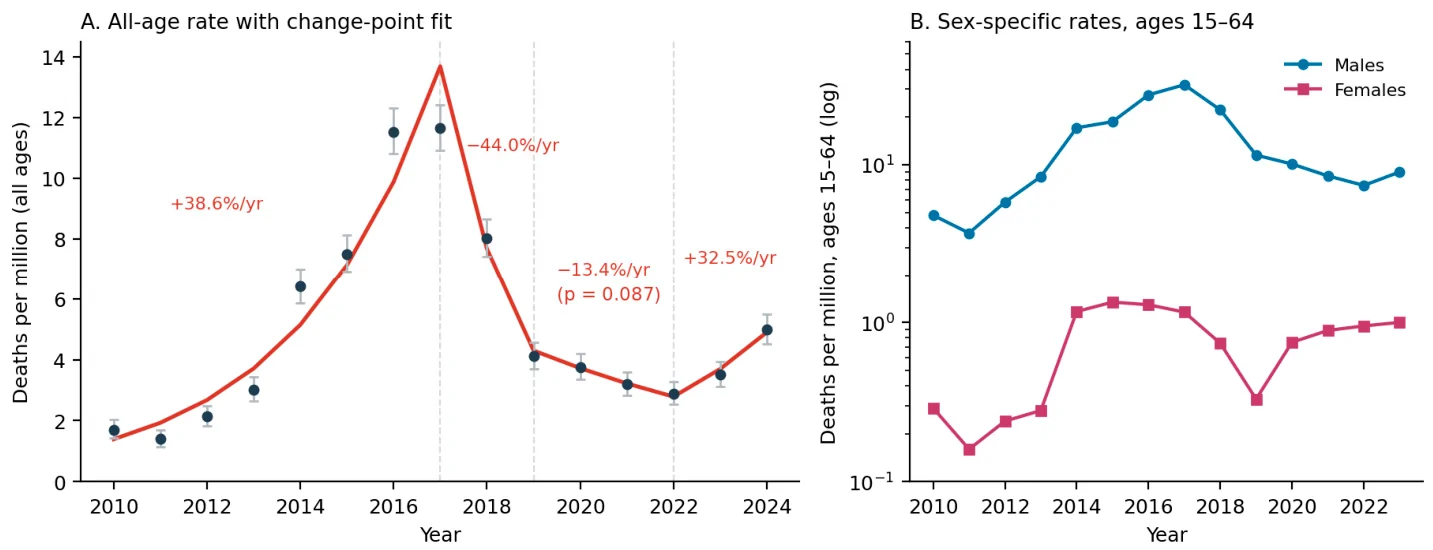
Direct drug-induced mortality in Türkiye. (**A**) All-age rate, 2010–2024: observed rates with exact Poisson 95% confidence intervals and the fitted change-point quasi-Poisson model (change points 2017, 2019 and 2022, dashed verticals); (**B**) sex-specific rates among persons aged 15–64, 2010–2023, as reported by the EUDA with Eurostat denominators (2023 values rounded to integers by the source).

A total of 6129 direct drug-induced deaths were recorded in Türkiye between 2010 and 2024. Annual deaths increased from 126 in 2010 to a peak of 941 in 2017, declined in each successive year to 246 in 2022, and then rose to 300 in 2023 and 427 in 2024. The 2024 count was 73.6% above the 2022 trough and 54.6% below the 2017 peak. The corresponding all-age mortality rate rose from 1.71 per million population in 2010 to 11.64 in 2017, fell to 2.88 in 2022, and returned to 4.98 by 2024 (Table 1; Figure 2).

In the change-point quasi-Poisson model, the series unfolded in four phases. Growth ran at +38.6% per year during 2010–2017 (95% CI +30.2 to +47.5), followed by a steep decline at −44.0% per year during 2017–2019 (95% CI −53.1 to −33.0), a shallow drift at −13.4% per year during 2019–2022 not distinguishable from a plateau (95% CI −26.9 to +2.5), and renewed growth at +32.5% per year during 2022–2024 (95% CI +2.0 to +72.3; *p* = 0.038).

### 3.2. Demographic Profile

Between 2010 and 2024, 94.7% of decedents with reported sex were male (Table 1). The female share fluctuated between 2.7% and 6.6% until 2019 with no trend, rose to 11.1% by 2022 (OR 1.55 per year over 2019–2022, 95% CI 1.28–1.86; *p* < 0.001), and fell back to 7.7% in 2023 and 6.3% in 2024 (OR 0.68 per year, 95% CI 0.49–0.96; *p* = 0.030). In absolute terms, female deaths numbered between 4 and 35 per year over 2010–2022, from 7 in 2010 to a maximum of 35 in 2015 and 27 in 2022, so all sex-specific trend estimates rest on small numbers. Sex-specific mortality rates diverged accordingly. Among persons aged 15–64, the male rate rose from 4.8 per million in 2010 to 32.0 in 2017 and fell to 7.4 by 2022, whereas the female rate never exceeded 1.34 per million; the male-to-female rate ratio widened from 17 in 2010 to 35 in 2019 and narrowed to 8 in 2022 (Figure 2B).

The age profile changed markedly over time and by 2022–2024 had returned close to its 2010 distribution. The share aged under 25 rose from 17.7% in 2010 to a peak of 35.7% in 2014 and returned to 18–19% in 2022–2024, while the share aged 45–64 fell from 16.9% in 2010 to a low of 7.8% in 2012 and recovered to 16–19% by 2022–2024 (Table 1). Over 2014–2024 the odds of a decedent being under 25 declined by 9% per year (OR 0.91, 95% CI 0.88–0.95; *p* < 0.001) and the odds of being aged 45–64 rose by 7% per year (OR 1.07, 95% CI 1.05–1.09; *p* < 0.001). Mean age at death followed the same course. It was about 34 years in 2010–2011 and 30–31 years during 2012–2019, and it rose after 2019 to 35.0 years in 2023 (0.69 years per calendar year, 95% CI 0.18–1.20). Deaths above age 64 were negligible throughout (≤1%).

### 3.3. Substance Profile of Drug-Induced Deaths

#### 3.3.1. Substance Involvement in All Deaths

The substances implicated in deaths turned over almost completely within the study period (Table 2A). Opioids were present in 76.5% of deaths in 2012, 31.7% in 2016 and 23.3% in 2017; on the EUDA measure the share was 36–37% in 2021–2022 and 15.5% in 2024, a measure that is broader than the earlier one, so the endpoint contrast understates the decline. Heroin, detected in 41–50% of deaths in 2012–2013, fell to 37.7% in 2019 and 14.0% in 2023. Synthetic cannabinoids followed the opposite course. Present in 4.3% of deaths in 2012 and 41.0% in 2016, they were detected in 59.9% in 2017, fell to 3.3% by 2022, and returned to 47.8% (204/427) in 2024. Methamphetamine, present in fewer than 5% of deaths in 2012–2013 and 6.2% in 2018, rose to 56.9% in 2022 before falling to 33.3% in 2024. MDMA involvement rose from 17.9% in 2012 to 40.4% in 2019 and fell to 18.7% in 2022 before partially recovering.

While the substances changed, the pattern of use did not. The polysubstance share fluctuated narrowly between 57.1% and 64.9% with no temporal trend (OR 0.99 per year, 95% CI 0.95–1.02; *p* = 0.433), and the single-substance share correspondingly showed none (35.1–42.9%).

#### 3.3.2. Single-Substance Deaths

**Figure 3:**
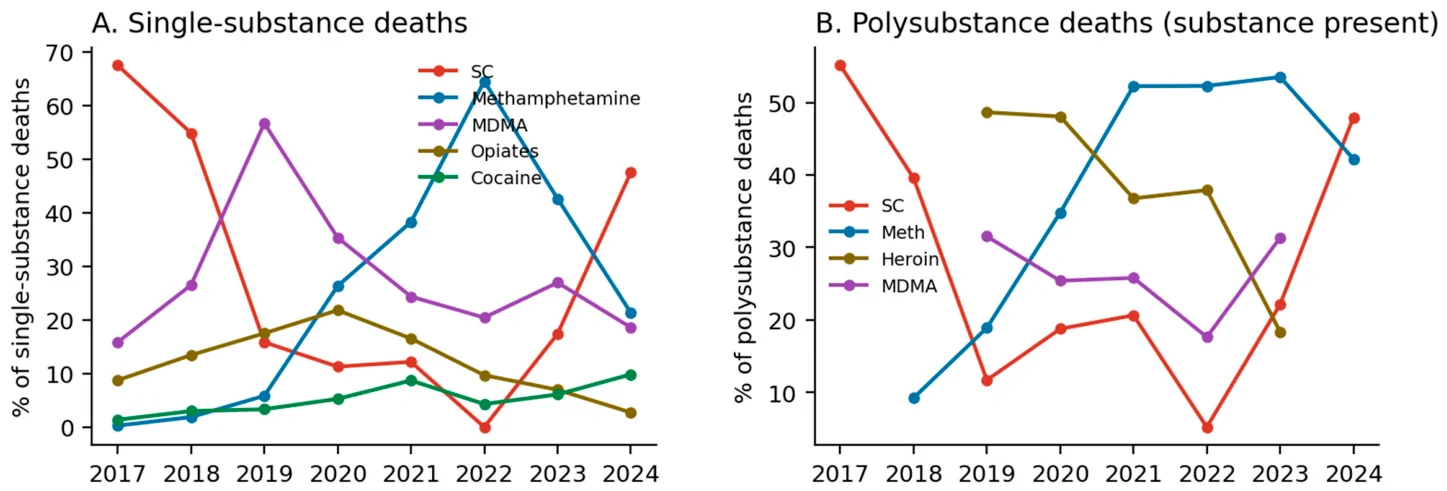
Substance composition of drug-induced deaths, 2017–2024. (**A**) Substance shares of single-substance (sole-cause) deaths. (**B**) Substance presence within polysubstance deaths (non-exclusive).

Single-substance attribution (the substance judged at autopsy to be solely responsible for death) shows the rotation more sharply (Table 2B; Figure 3). The leading sole cause changed three times, from synthetic cannabinoids in 2017–2018, to MDMA in 2019–2020, to methamphetamine in 2021–2023, and to synthetic cannabinoids again in 2024. The composition differed significantly across 2019–2024 and between the endpoint years (χ^2^(15) = 224.5 and χ^2^(3) = 67.2; both *p* < 0.001). MDMA was already a leading single substance before the itemised series began. In 2015, 62 deaths were attributed to MDMA alone and 63 to synthetic cannabinoids alone (Table 2, footnote c), and in 2019 MDMA alone accounted for 56.7% of single-substance deaths (68/120). Sole-cause opiate deaths declined from 36 in 2018 to 5 in 2024. Sole-cause cocaine deaths reached their series maximum in 2024 (18 deaths; 9.8% of single-substance cases).

#### 3.3.3. Substance Involvement in Polysubstance Deaths

Within polysubstance deaths, the composition rotated in parallel (Table 2C; Figure 3B). Synthetic cannabinoids were present in 55.1% in 2017 and 39.6% in 2018. Among the substances reported thereafter, heroin was the most frequent component in 2019–2020 (48.6% and 48.1%), methamphetamine in 2021–2023 (52.3–53.5%), and synthetic cannabinoids in 2024 (48.0%, overtaking methamphetamine at 42.2%). Specific substance combinations are not reported in the source and could not be analysed.

#### 3.3.4. The 2017 Peak and the 2024 Increase

Total deaths rose from 300 in 2023 to 427 in 2024 (rate ratio 1.42, 95% CI 1.23–1.65; *p* < 0.001). The increase was confined to the recorded synthetic cannabinoid component. Deaths with synthetic cannabinoid involvement more than tripled (61 to 204; RR 3.34, 95% CI 2.51–4.45; *p* < 0.001), whereas deaths without synthetic cannabinoid involvement did not change (239 to 223; RR 0.93, 95% CI 0.78–1.12; *p* = 0.457), and methamphetamine-involved deaths were likewise stable (148 to 142; RR 0.96, 95% CI 0.76–1.21; *p* = 0.726). In additive terms, the net national increase of 127 deaths is more than fully accounted for by the increase of 143 synthetic cannabinoid-involved deaths. Among 2024 deaths in which each substance was detected, synthetic cannabinoids were recorded as the single substance more often than methamphetamine (42.6%, 87/204, versus 27.5%, 39/142).

**Figure 4:**
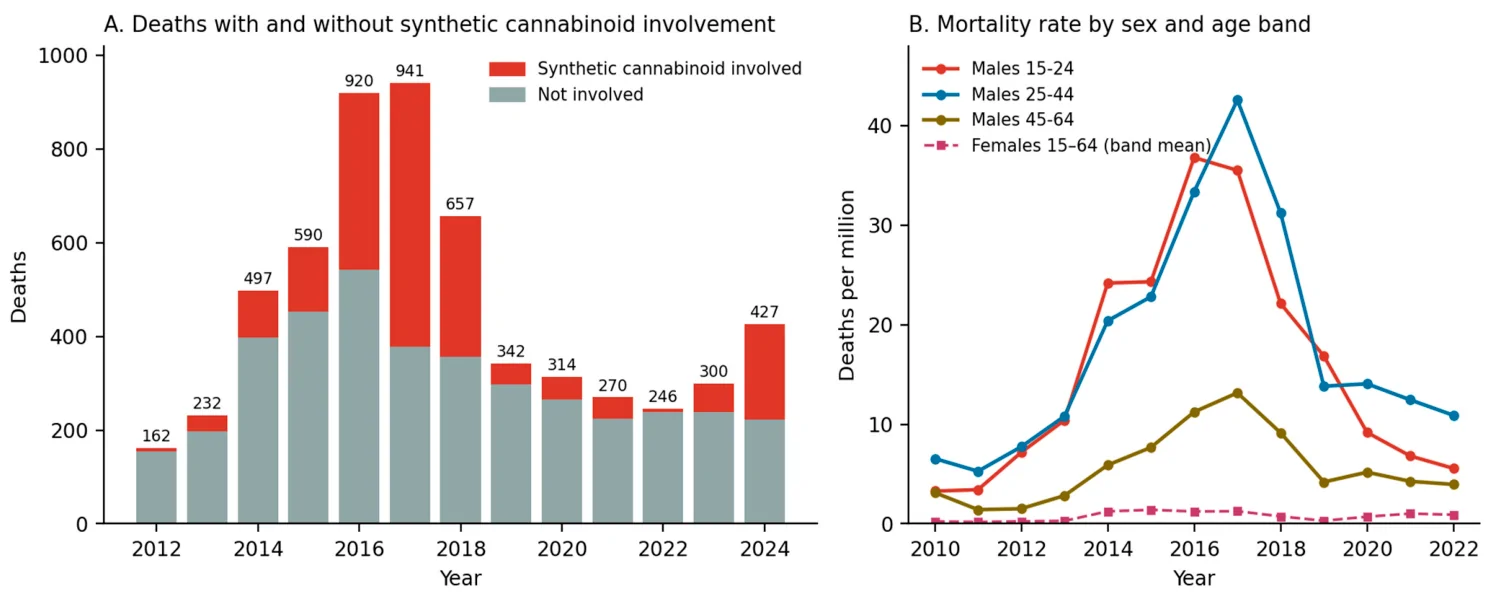
Synthetic cannabinoid involvement and age- and sex-specific mortality trends in Türkiye. (**A**) Deaths with and without synthetic cannabinoid involvement, 2012–2024. (**B**) Mortality rates per million by sex and age band, 2010–2022, from the sex- and age-specific counts reported to the EUDA (Eurostat denominators).

Extending the decomposition to the full period places 2024 against the earlier peak (Figure 4A). Deaths without synthetic cannabinoid involvement rose from 197 in 2013 to 543 in 2016, then fell to 377 in 2017, 297 in 2019 and 223 in 2024. Deaths with synthetic cannabinoid involvement rose from 35 in 2013, to 377 in 2016, and 564 in 2017; fell to 8 by 2022; and returned to 204 in 2024. Between 2016 and 2017 the two components moved in opposite directions of a similar size (synthetic cannabinoid-involved deaths +187, other deaths −166) while the total changed by 21. By age, the rise and fall were concentrated among men under 45. The male rate at ages 15–24 rose from 3.3 per million in 2010 to 36.8 in 2016, and at ages 25–44 from 6.5 to 42.6 in 2017, before both fell below 12 by 2022, whereas the male rate at ages 45–64 peaked at 13.2 and the female rate never exceeded 1.3 (Figure 4B).

### 3.4. Türkiye in the European Context

In 2024 the EUDA recorded a minimum of 7589 drug-induced deaths in the EU, corresponding to 25.0 per million population aged 15–64, against 7.20 per million in Türkiye (95% CI 6.53–7.92; Supplementary Table S2). Applying the EU rate to the Turkish population aged 15–64 yields an observed-to-expected ratio of 0.288 (422 observed against 1465.7 expected; 95% CI 0.261–0.317). Direct standardisation to the comparator panel (below) gives an age-standardised rate ratio of 0.15 for 2022, the most recent year available.

**Figure 5:**
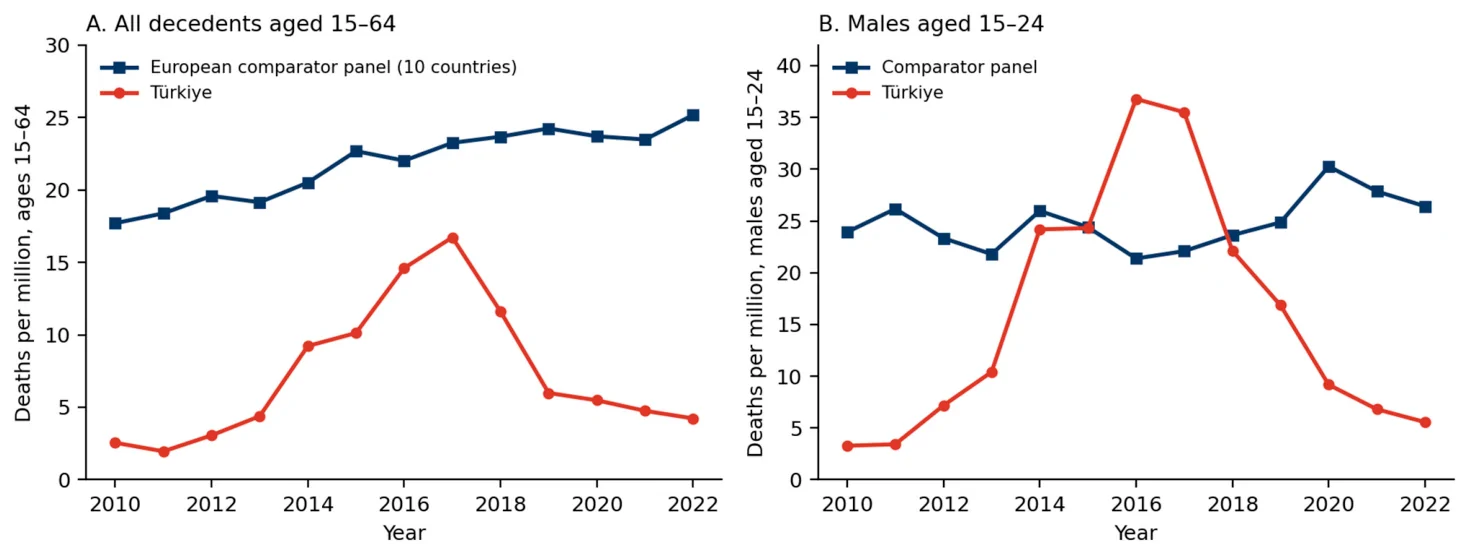
Drug-induced mortality rates in Türkiye and a fixed European comparator panel, 2010–2022, per million population. (**A**) Persons aged 15–64. (**B**) Males aged 15–24. The panel comprises the ten EU countries with complete sex- and age-specific data for every year (Supplementary Table S3). Data: EUDA source tables (deaths reported under the drug-related deaths protocol; Eurostat denominators).

Year-by-year comparison used the fixed ten-country panel defined in the Methods (Supplementary Table S3). The panel rate among persons aged 15–64 rose steadily from 17.7 per million in 2010 to 25.2 in 2022, close to the EUDA estimate for the whole EU (22.5). The Turkish rate on the same basis rose from 2.6 per million in 2010 to 16.7 in 2017 and fell to 4.2 in 2022 (Figure 5A). The ratio of the Turkish rate to the panel rate therefore rose from 0.14 to 0.72 and fell back to 0.17; after direct standardisation to the panel’s age and sex structure, the ratio was 0.14, 0.64 and 0.15, respectively. In a segmented regression of the rate ratio at the 2017 and 2019 change points, the ratio grew by 33.8% per year during 2010–2017, fell by 43.3% per year during 2017–2019, and fell by 12.9% per year during 2019–2022 (Supplementary Methods). When all countries with complete data in a given year were pooled instead of the fixed panel (16 to 24 countries; Supplementary Table S3), the ratio followed the same course, from 0.17 in 2010 to a peak of 0.90 in 2016 and back to 0.17 in 2022, running slightly above the panel ratio throughout (median difference 0.06) because the pool partly includes large lower-rate countries; the fixed panel is therefore the conservative comparator.

The comparison differs by age. Among males aged 15–24, the panel rate was stable at 21 to 30 per million throughout, whereas the Turkish rate exceeded it by a factor of 1.6 to 1.7 in 2016–2017 before falling to 5.6 per million in 2022 (Figure 5B). In the broader pool of all countries with complete data, the Turkish rate among young males in 2016 was 2.8 times the European rate (36.8 versus 13.2 per million).

**Figure 6:**
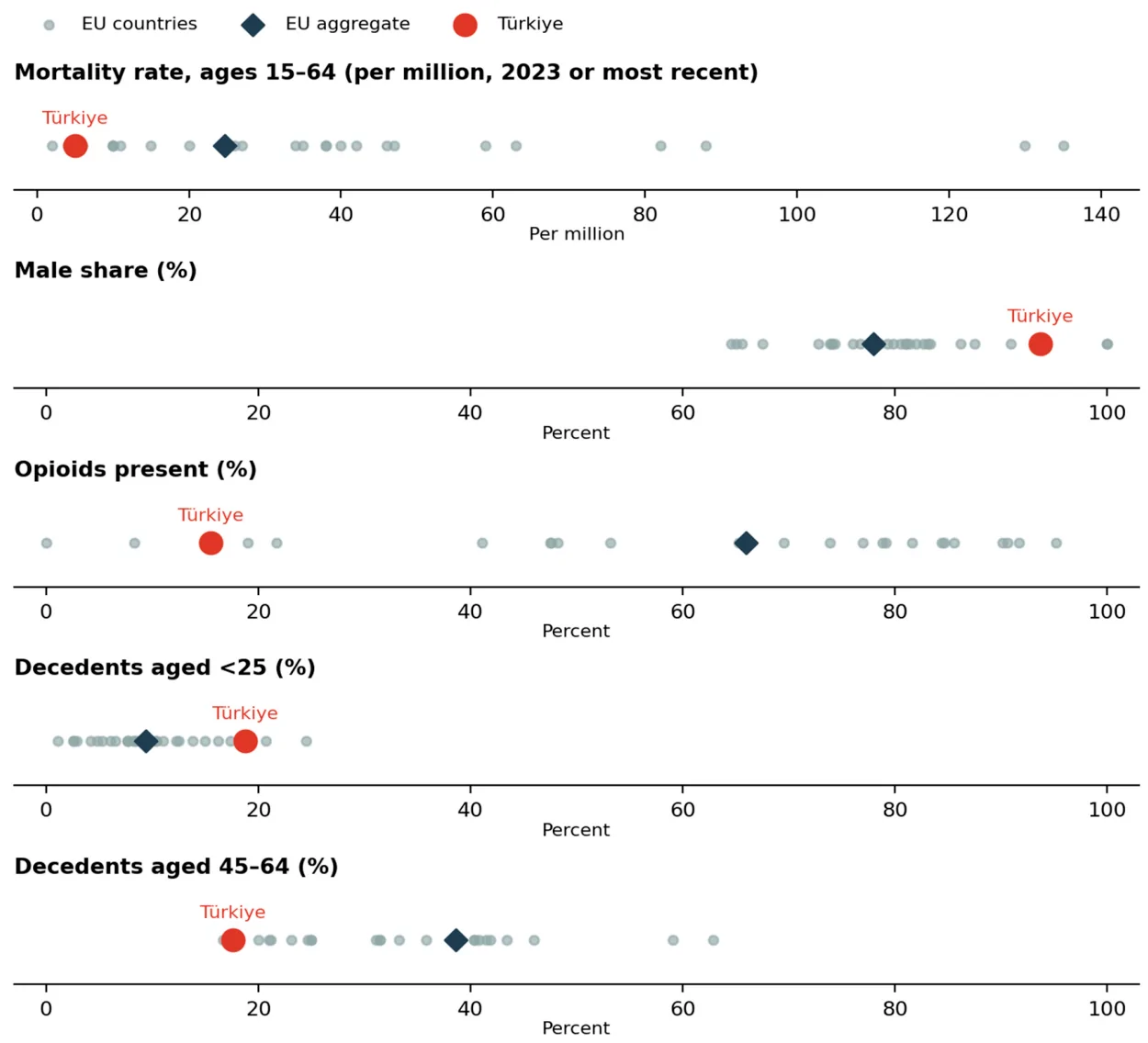
Türkiye against the European distribution. Top strip: drug-induced mortality rate among persons aged 15–64, 2023 or most recent year, with the EU estimate of 24.7 per million (EUDA 2025 source tables; Türkiye 5 per million, values rounded by the source). Lower strips: male share, opioid involvement and age distribution of decedents, 2024 or most recent year (European Drug Report 2026 source tables). Grey dots: individual EU countries; diamond: EU aggregate; and red: Türkiye.

On each demographic indicator, Türkiye lay at or near one end of the European distribution (Figure 6). Males accounted for 93.7% of decedents in Türkiye against an EU aggregate of 78.0% (OR 4.18, 95% CI 2.83–6.17, treating the EU value as fixed), the third-highest male share among 29 reporting countries, exceeded only by Malta and Cyprus (both 100%, small caseloads). Opioids were present in 15.5% of deaths in Türkiye in 2024, the third-lowest share among 28 countries, above only Cyprus (0%) and Malta (8.3%), versus 66.0% in the EU aggregate (Table 2A). The age structure was inverted relative to Europe. In Türkiye, 18.8% of decedents were under 25 (EU-country median 8.3%), and 62.7% were aged 25–44 (median 52.1%), whereas 17.6% were aged 45–64 (median 31.5%), and 0.9% were 65 or older (median 3.8%).

Substance involvement also differed. Heroin was involved in about one fifth of EU deaths in 2024, against 14.0% in Türkiye in 2023, the most recent year published. Around one quarter of EU deaths involved cocaine. In Türkiye, cocaine was recorded as a sole cause in 18 deaths (4.2%) in 2024; its presence in polysubstance deaths is not reported. Methadone, reported in 31–42% of deaths in several EU countries, is not listed among drug-induced deaths in Türkiye. Synthetic cannabinoids, present in 47.8% of deaths in Türkiye in 2024, are not among the principal substances in EU-level mortality reporting, where the synthetic substances highlighted are highly potent opioids such as the nitazenes. The substances most frequently implicated therefore differed. In the EU they were opioids, including methadone and potent synthetic opioids. In Türkiye they were synthetic cannabinoids and methamphetamine.

Polysubstance involvement cannot be benchmarked numerically. The EUDA describes polysubstance toxicity as the norm across Europe but publishes no aggregate share to set against the Turkish figure (57–65%). The composition of polysubstance deaths nonetheless differed. In EU countries polysubstance deaths most often involved opioids, with cocaine detected mostly in their presence and benzodiazepines combined with opioids in most deaths in six countries. In Türkiye, the most frequent components of polysubstance deaths were methamphetamine in 2021–2023 and synthetic cannabinoids in 2024, with opioids present in a declining minority (Table 2C).

On each of the three structural indicators (male share, opioid involvement and age distribution), Türkiye ranked within the three countries furthest from the EU aggregate. Among countries reporting more than 70 deaths, it was the only one to do so on all three. Türkiye’s position on each indicator was essentially unchanged in every year from 2019 to 2024 (Supplementary Figure S1).

## 4. Discussion

### 4.1. Principal Findings

Drug-induced deaths in Türkiye rose seven-fold between 2010 and 2017, fell by almost two thirds in the following two years, drifted downward to 2022 and have since risen again. The wave was carried by men under 45. At its height the mortality rate among young men in Türkiye exceeded that of a European comparator panel by a factor of 1.6 to 2.8, while the all-ages rate remained below the European level. Over the same fifteen years, the substances implicated turned over almost completely. Opioids fell from three quarters of deaths to one sixth, and the leading substance changed four times, from heroin to synthetic cannabinoids, to MDMA, to methamphetamine and back to synthetic cannabinoids. The increase of 2024 was confined to deaths recorded as involving synthetic cannabinoids. Throughout, Türkiye combined one of the lowest mortality rates in Europe with decedents who were among the most male, the youngest and the least opioid-involved of any reporting country.

### 4.2. The Rise to 2017 and Its Subsequent Reversal

Turkish and European reports have described the 2017 peak primarily as a synthetic cannabinoid phenomenon [5,25]. However, the rise to 2017 was not confined to a single substance. Between 2013 and 2016, deaths without synthetic cannabinoid involvement almost tripled, from 197 to 543. Deaths involving opioids, MDMA and amphetamines also increased substantially. Market indicators moved in the same direction. Recorded synthetic cannabinoid incidents increased sharply, Türkiye seized the second-largest quantity of MDMA worldwide in 2017, and methamphetamine entered the market during the same period [1,17,18]. The 2017 peak therefore followed a broader expansion in drug-related mortality, on which a large synthetic cannabinoid component was superimposed.

Expanding analytical coverage may nevertheless have amplified the recorded increase in synthetic cannabinoid involvement. In 2013, synthetic cannabinoids were analysed in only four of the CFM’s ten laboratories, and the method covered three compounds [26]. Detecting new psychoactive substances depends on the compounds included in analytical panels and the availability of reference standards, both of which must be updated as the market changes [4,13,14]. The change between 2016 and 2017 is consistent with improved substance attribution. Total deaths increased by only 21 between 2016 and 2017, whereas deaths involving synthetic cannabinoids increased by 187 and deaths without synthetic cannabinoid involvement decreased by 166. The extent to which this divergence reflects improved toxicological detection cannot be quantified from the published data.

Expanded analytical capacity may have contributed to the post-2017 decline through a pathway other than measurement. More complete and timely identification of synthetic cannabinoids could have strengthened early warning, scheduling, supply-control responses and clinical recognition, thereby potentially reducing exposure and fatal risk. This pathway cannot be evaluated using the published aggregate mortality data, because the reports do not provide annual panel coverage, update lags or information on how toxicological findings informed subsequent interventions. Evaluating whether improved detection contributed to the decline would require a separate study linking changes in analytical coverage and case-level toxicology findings with surveillance and policy responses. The concurrent decline in total deaths and deaths without synthetic cannabinoid involvement indicates that the broader national trend was not simply a change in substance attribution.

### 4.3. A Young Men’s Wave, Not an Ageing Cohort

European overdose mortality is the mortality of an ageing cohort. Deaths at ages 50 to 64 in the EU more than doubled between 2013 and 2023 [8,27]. In the United Kingdom and Australia, mortality risk among long-term users rises with age [28,29]. Türkiye does not fit this pattern. Its wave rose and fell among men aged 15 to 44; the rate among men aged 45 to 64 never exceeded 13 per million, and the share of decedents under 25 rose from 18% to 36% and returned to 18%. The cohort in Türkiye did not age. The wave passed through it and left the age structure where it had been.

Why young men were exposed is not explained by the mortality data alone, but the market and policy context suggests contributing conditions. Synthetic cannabinoid products were cheap, sold as herbal preparations and widely perceived as natural. Their harms appeared first in emergency departments, among overwhelmingly young men [15,16]. Beyond price and perception, the structure of drug testing under probation may also have played a part. People found in possession of drugs for use are placed under probation with periodic urine testing (Penal Code Article 191), with more than 167,000 such orders in 2014 [1]. In an English prison, Ralphs et al. [30] found that avoidance of detection in mandatory drug tests was the primary motivation for synthetic cannabinoid use. Routine screening relies on immunoassay panels, which during the years of the wave either did not include synthetic cannabinoids or covered only first-generation compounds while the market had moved on to newer analogues [31,32]. As a contextual hypothesis rather than a finding of this study, a comparable incentive may have operated among the young men who are the principal subjects of probation testing; the hypothesis is untested but testable.

### 4.4. Sex Differences and the Small Number of Women

Women accounted for a very small share of deaths throughout the period; their share was lowest during the peak years and never above 11.1%. Men accounted for 95% of decedents in Türkiye over the period, against 78% in the EU [8]. Part of this gap is real exposure. Past-year cannabis use among young adults was 0.2% in women and 3.5% in men [33]. Men made up 95–97% of addiction treatment admissions [1,34]. The sex gap in deaths is of the same order as the sex gap in use and in treatment. A smaller part of the gap may reflect deaths that never reach the forensic system. Clinicians in Türkiye describe women who use drugs as subject to a specific moral stigma, in which drug use is seen as a betrayal of the role assigned to women. They attribute the scarcity of data on women to under-reporting for that reason [35].

The rise in the female share to 11% by 2022 reflected mainly the steep fall in deaths among men, with a smaller absolute increase among women. That increase occurred in the years when methamphetamine dominated the deaths, and in other countries the share of women among decedents has been higher in stimulant-related than in opioid-related mortality [28]. Whether the same holds in Türkiye cannot be verified, because substance involvement is not published separately for men and women. Harms among women also extended beyond the deaths counted here. In a previous CFM study, we reported 28 fetal deaths with methamphetamine detected in fetal blood between 2020 and 2024 [36]. Many involved pregnancies without prenatal care and fetal expulsion or discovery at home. Intent is also not recorded, although across Europe overdose deaths among women more often involve suicidal intent [27]. Women make up one in twenty treatment admissions, and no women-specific services exist [37]. Services whose admissions are almost entirely male are unlikely to reach the women at risk.

### 4.5. The Shift from Opioids to Synthetic Cannabinoids and Stimulants

In North America and much of Western Europe, stimulants have entered overdose mortality alongside opioids. The fourth wave of the North American crisis is defined by fentanyl–stimulant co-involvement [11,38,39], and in Europe cocaine is detected mostly in deaths that also involve opioids [8]. In Türkiye the stimulants and the synthetic cannabinoids did not join the opioids. They replaced them. Opioids were present in three quarters of deaths in 2012 and in one sixth in 2024, deaths attributed to opiates alone fell from 36 to 5, and polysubstance deaths came to be anchored on methamphetamine rather than heroin. McAuley et al. [40] described two profiles of new psychoactive substance deaths in Scotland: an older opioid-combining group and a younger stimulant-combining group. Türkiye consists almost entirely of the second.

Most of the heroin seized in Türkiye is in transit to Western Europe rather than destined for domestic use [1]. The domestic market moved instead toward synthetic cannabinoids and stimulants, which were cheaper than heroin; easier to obtain; and, until each compound was scheduled, not illegal [3,15]. At the same time, methadone is not reported among drug-induced deaths in Türkiye [8]. Opioid agonist treatment in Türkiye has relied on buprenorphine–naloxone since 2010 [19]. The absence of methadone-related deaths is the mirror image of a treatment system built on a different agonist, and a reminder that the substance profile of overdose deaths reflects the treatment system as well as the illicit market.

### 4.6. Implications for Surveillance and Prevention

The Turkish experience tests a supply-led response against a fast-moving synthetic market. The legislative changes of 2014, which raised sentences for synthetic cannabinoid offences by half and criminalised use [3], preceded the mortality peak by three years and did not prevent it. The scheduling of 125 compounds between 2011 and 2014 did not interrupt the turnover of analogues. Synthetic cannabinoids returned in 2023 and 2024 to account for half of all deaths, and provisional data for 2025 reported by the EUDA point to a further increase [8]. These are observations at the level of years, not of individuals; whether enforcement contributed to the decline after 2017 cannot be determined from these data. What the record does show is that a decade of severe penalties and rapid scheduling was not sufficient to prevent the return of the same substance class among a new generation of young men.

In Türkiye, naloxone and agonist treatment would address the one death in six that involves an opioid. Harm reduction services monitored by the EMCDDA, including take-home naloxone and needle and syringe programmes, were reported as unavailable in Türkiye [41]. Deaths caused by synthetic cannabinoids and stimulants require a different set of responses. Türkiye already operates an early warning group that schedules new compounds [1]. Linking the laboratory analysis of seized drugs to post-mortem toxicology in near real time would turn a scheduling instrument into a public health one [42]. Drug checking services would identify which synthetic cannabinoid is in circulation before deaths accumulate [43]. Stimulant dependence would be treated with the psychosocial approaches that are the only established options [9,44]. Prevention resources should follow the age profile of the deaths. In Europe that profile points to ageing opioid users; in Türkiye it points to men under 25 and the settings where they are found, including schools, military service and probation. Which preventive approaches work for synthetic cannabinoids among young men is an open question, and programmes adopted from other substances should be evaluated rather than assumed to be effective. The CFM already produces the toxicological data; what is needed is a public health system that turns them into prevention and early warning.

Screening panels, in probation testing and elsewhere, should cover the synthetic cannabinoids actually in circulation, and their coverage and update lag should be published. This recommendation follows directly from the 2016–2017 attribution shift and from the absence of published panel-coverage records. The monitoring system should also report what is needed to interpret its series. Annual rates rather than counts, fixed age bands, substances by sex, substance combinations and the analytical coverage of each laboratory would allow future changes to be read as changes in mortality or in detection. Each of these items corresponds to a gap encountered in assembling the present dataset. A cross-check against the cause-of-death statistics of the Turkish Statistical Institute, as the European protocol recommends [20], would settle the question for the years already recorded. Of these recommendations, those concerning reporting, screening coverage and the mortality cross-check follow directly from the data gaps documented in this study; the service responses rest on the substance and age profile of the deaths; and the preventive approaches require evaluation before adoption.

### 4.7. Strengths and Limitations

This is the first analysis to bring together the deaths recorded in Türkiye over fifteen years across substances, sex and age. All published figures trace back to a single producer, the CFM, and the reports that carry them proved identical where they overlapped. Rates are based on real denominators, and the European comparison uses a fixed panel with direct sex- and age- standardisation. Several limitations follow from the nature of the data. The analysis is ecological, and associations between market or policy events and mortality are not causal [45]. The national time series has fifteen annual observations, so the trend estimates for the periods between change points rest on only a few years each [46]. The analytical coverage of synthetic cannabinoid testing, and how it changed over the period, could not be measured. The sex- and age-specific counts reported for 2014 to 2017 exclude cases whose age or sex was not recorded, up to 141 cases in 2016, so the rates for those years are slightly understated. Extreme-assignment bounds for these cases are reported in the Supplementary Methods; the male predominance and the young age structure hold even at the implausible extremes. Blood concentrations are often unavailable in the deaths reported from Türkiye [18], and substance combinations within polysubstance deaths are not published. The relative contributions of stimulants, opioids and synthetic cannabinoids to polysubstance mortality therefore cannot be compared directly: component shares describe how often each substance was present, not which combinations were lethal, and a substance frequently present as a component may rarely have been the decisive agent. Trends in autopsy referral are a further source of uncertainty. Referral statistics are not published, so a change in referral practice cannot be excluded as a contributor to the recorded trends, although the sharp improvement in the completeness of age and sex recording in 2018 argues against weakening case ascertainment during the years of decline. Finally, the findings on women rest on small numbers.

## 5. Conclusions

Drug-induced mortality in Türkiye remains low by European standards, but its course has been highly unstable. Over fifteen years, deaths rose seven-fold to a peak in 2017, fell by almost two thirds, and began to rise again after 2022. The net increase in 2024 was confined to deaths recorded as involving synthetic cannabinoids. Türkiye also differs from the dominant European mortality profile. Compared with Europe, decedents are younger and more often male, women account for a far smaller share of deaths, and opioids appear in one death in six against two in three in the EU. Synthetic cannabinoids and stimulants, rather than opioids, have dominated recent mortality. Expanding analytical capacity may have both improved ascertainment of synthetic cannabinoid involvement and strengthened the surveillance and control responses that followed the 2017 peak. A response built primarily around opioids can address only a minority of drug-induced deaths in Türkiye. The findings point to the need for surveillance capable of distinguishing changes in mortality from changes in detection, together with prevention and services directed at synthetic cannabinoids, stimulants and the young men most at risk.

## Figures and Tables

**Table 1:** Direct drug-induced deaths, crude mortality rates and decedent characteristics, Türkiye, 2010–2024.

Year	Deaths	Rate per Million (95% CI)	Female (%)	Mean Age, y	<25 (%)	25–44 (%)	45–64 (%)	Rate, Males (15–64)	Rate, Females (15–64)
2010	126	1.71 (1.42–2.03)	5.6	34.2	17.7	65.3	16.9	4.8	0.29
2011	105	1.41 (1.15–1.70)	4.1	34.0	24.7	63.9	11.3	3.7	0.16
2012	162	2.14 (1.82–2.50)	3.9	30.2	31.2	61.0	7.8	5.8	0.24
2013	232	3.03 (2.65–3.44)	3.1	31.1	31.2	58.9	9.8	8.4	0.28
2014	497	6.40 (5.85–6.98)	6.3	–	35.7	54.3	10.0	17.1	1.17
2015	590	7.49 (6.90–8.12)	6.6	31	32.6	55.5	11.8	18.7	1.34
2016	920	11.53 (10.79–12.30)	4.4	31	32.3	55.5	12.2	27.6	1.29
2017	941	11.64 (10.91–12.41)	3.4	32.0	27.6	59.9	12.6	32.0	1.16
2018	657	8.01 (7.41–8.65)	3.1	32.6	24.2	63.3	12.6	22.2	0.74
2019	342	4.11 (3.69–4.57)	2.7	30.9	34.2	54.4	11.4	11.5	0.33
2020	314	3.76 (3.35–4.19)	6.8	33.5	21.4	62.8	15.9	10.1	0.75
2021	270	3.19 (2.82–3.59)	9.3	33.4	21.2	62.8	16.0	8.5	0.89
2022	246	2.88 (2.54–3.27)	11.1	34.0	18.1	65.8	16.0	7.4	0.95
2023	300	3.51 (3.13–3.94)	7.7	35.0	19.2	61.6	19.2	9	1
2024	427	4.98 (4.52–5.48)	6.3	34.7	18.8	62.7	17.6	–	–

Deaths and rates: national all-age counts (Turkish Drug Reports 2018–2025; European Drug Report 2026 source tables for 2010–2012) per 1,000,000 population (Turkish Statistical Institute), with exact Poisson 95% confidence intervals. Female share, age distribution and sex-specific rates for 2010–2022: derived from the counts of deaths at ages 15–64 by sex and age band that Türkiye reports to the EMCDDA/EUDA, with Eurostat denominators [24]; for 2014–2017 these counts fall short of the national totals by 18, 57, 141 and 34 deaths (cases with unrecorded age or sex). 2023: EUDA 2025 tables (deaths with known age; rates rounded to integers by the source); 2024: national report (female share), and European Drug Report 2026 (age distribution); sex-specific rates not yet published. Mean age: 2014 Reitox national report (2010–2013), EMCDDA Country Drug Reports (2015–2016, rounded), and national reports (2017–2024); not available for 2014. Deaths and populations aged 15–64 are given in Supplementary Table S2.

**Table 2:** Substance involvement in all deaths, 2012–2024 (Panel (**A**)); mutually exclusive single-substance classification, 2017–2024 (Panel (**B**)—single-substance (sole-cause) deaths, *n* [% of single-substance total]); and substance involvement within polysubstance deaths, 2017–2024 (Panel (**C**)—*n* (% of polysubstance total)).

(**A**)							
**Year**	**Deaths, *n***	**Synthetic Cannabinoids**	**Methamphetamine**	**Heroin**	**MDMA**	**Any Opioid**	
2012 ^a^	162	7 (4.3)	1 (0.6)	67 (41.4)	29 (17.9)	124 (76.5)	
2013 ^a^	232	35 (15.1)	11 (4.7)	115 (49.6)	58 (25.0)	164 (70.7)	
2014 ^b^	497	99 (19.9)	–	–	–	286 (57.5)	
2015 ^c^	590	137 (23.2)	–	–	166 (28.1)	315 (53.4)	
2016 ^c^	920	377 (41.0)	–	–	–	292 (31.7)	
2017	941	564 (59.9)	–	–	–	219 (23.3)	
2018	657	301 (45.8)	41 (6.2)	–	–	–	
2019	342	45 (13.2)	49 (14.3)	129 (37.7)	138 (40.4)	–	
2020	314	49 (15.6)	98 (31.2)	116 (36.9)	93 (29.6)	–	
2021	270	46 (17.0)	125 (46.3)	76 (28.1)	68 (25.2)	36 ^d^	
2022	246	8 (3.3)	140 (56.9)	67 (27.2)	46 (18.7)	37.4 ^d^	
2023	300	61 (20.3)	148 (49.3)	42 (14.0)	89 (29.7)	–	
2024	427	204 (47.8)	142 (33.3)	–	–	15.5 ^d^	
(**B**)							
**Year**	**Single Total**	**Synthetic Cannabinoids**	**Methamphetamine**	**MDMA**	**Opiates**	**Cocaine**	**Other ^e^**
2017	366	247 (67.5)	1 (0.3)	58 (15.8)	32 (8.7)	5 (1.4)	23 (6.3)
2018	268	147 (54.9)	5 (1.9)	71 (26.5)	36 (13.4)	8 (3.0)	1 (0.4)
2019	120	19 (15.8)	7 (5.8)	68 (56.7)	21 (17.5)	4 (3.3)	1 (0.8)
2020	133	15 (11.3)	35 (26.3)	47 (35.3)	29 (21.8)	7 (5.3)	0
2021	115	14 (12.2)	44 (38.3)	28 (24.3)	19 (16.5)	10 (8.7)	0
2022	93	0 (0.0)	60 (64.5)	19 (20.4)	9 (9.7)	4 (4.3)	1 (1.1)
2023	115	20 (17.4)	49 (42.6)	31 (27.0)	8 (7.0)	7 (6.1)	0
2024	183	87 (47.5)	39 (21.3)	34 (18.6)	5 (2.7)	18 (9.8)	0
(**C**)							
**Year**	**Polysubstance Total**	**Synthetic Cannabinoids**	**Methamphetamine**	**Heroin**		**MDMA**	
2017	575	317 (55.1)	–	–		–	
2018	389	154 (39.6)	36 (9.3)	–		–	
2019	222	26 (11.7)	42 (18.9)	108 (48.6)		70 (31.5)	
2020	181	34 (18.8)	63 (34.8)	87 (48.1)		46 (25.4)	
2021	155	32 (20.6)	81 (52.3)	57 (36.8)		40 (25.8)	
2022	153	8 (5.2)	80 (52.3)	58 (37.9)		27 (17.6)	
2023	185	41 (22.2)	99 (53.5)	34 (18.4)		58 (31.4)	
2024	244	117 (48.0)	103 (42.2)	–		–	

Panel (**A**) percentages use annual deaths as denominators; categories are not mutually exclusive. ^a^ 2012–2013: Council of Forensic Medicine data reported in the 2014 Reitox national report (heroin = 6-monoacetylmorphine detected; any opioid = opiates present). ^b^ 2014: Council of Forensic Medicine national series as published in EMCDDA [5]. ^c^ 2015–2016: synthetic cannabinoid counts from the European Drug Report 2017 (137, of which 63 were with synthetic cannabinoids alone) and from the 2017 harms workbook as compiled by López-Pelayo et al. [4] (377); MDMA 2015 from the European Drug Report 2017 (166, of which 62 were alone); and opioid counts for 2014–2017 from the Council of Forensic Medicine series in EMCDDA [5]. ^d^ Percentages of deaths with any opioid present as reported to the EUDA (2021, 2022, 2024). Panel (**B**) categories are mutually exclusive and sum to the annual single-substance totals. ^e^ ‘Other’ comprises deaths attributed to substances not separately itemised for 2017 (*n* = 23, largely volatile substances), and single benzodiazepine-only (2018, 2019) and amphetamine-only (2022) deaths. Panel (**C**) values are derived as substance presence (Panel (**A**)) minus sole-cause deaths (Panel (**B**)); categories are not mutually exclusive.

## Data Availability

The compiled dataset (JCM_DRD_dataset_v1_1.xlsx, with every value labelled by its source publication), the complete Python code for data processing, statistical modelling and figure generation (analysis.py), and its model outputs (model_outputs.csv) are provided as Supplementary Materials with this article. All underlying values derive from publicly available official publications, itemised in Supplementary Table S1.

## Supplementary Materials

The following supporting information can be downloaded at https://www.mdpi.com/article/10.3390/jcm15218517/s1, Table S1: Variables, definitions, years of availability and sources; Table S2: Population denominators, Türkiye, 2010–2024; Table S3: European comparator panel: composition, pooled mortality rates and Türkiye-to-panel rate ratios, 2010–2022; Figure S1: Türkiye against the European distribution, 2019–2024; Supplementary Methods: Analytical coverage; S-M1: Consistency of the data across publication channels; S-M2: Values obtained from a published chart; S-M3: Change-point selection and fixed-knot sensitivity analysis; S-M4: Sensitivity to cases with unrecorded age or sex. References cited in the Supplementary Materials are included in the main reference list [2,5,8,14,15,21,23,24,25,26,31,32].
