# Supplementary material for: Drug-Induced Deaths in Türkiye, 2010–2024: Trends, Demographic and Substance Characteristics, and Comparison with Europe
Source: J Clin Med. 2026 Nov 2;15(21):8517. doi: 10.3390/jcm15218517 (PMC13763697; doi:10.3390/jcm15218517)
Supplement: Supplementary file 1 [file jcm-15-08517-s001.zip › Supplementary_Material.pdf]

Supplementary material

Drug-induced deaths in Türkiye, 2010–2024: trends, demographic and substance characteristics, and comparison with Europe

Supplementary Table S1

Variables, definitions, years of availability and sources.

Variable	Definition	Years	Source
Deaths, all ages	Direct drug-induced deaths, national total	2010–2024	TDR 2018–2025; EUDA table 7 (2010–2012)
Deaths, ages 15–64	National count designated for European reporting	2017–2024	TDR 2018–2025
Deaths by sex and age band (15–24, 25–44, 45–64)	Counts reported under the DRD protocol; Eurostat populations	2010–2022; 2023	EUDA FAQ 2024, table 15; FAQ 2025, figures 1-5, 1-8
Age distribution (<25, 25–44, 45–64, 65+)	Shares of deaths with known age	2023; 2024	EUDA source tables, EDR 2025, 2026
Mean age at death	Arithmetic mean, all ages	2010–2013; 2015–2024	NR2014; CDR 2017–2018; TDR
Substance presence	Deaths with the substance detected (non-exclusive)	2012–2013; 2014; 2015–2016; 2017–2024	NR2014; EMCDDA (2019); EDR 2017, López-Pelayo et al. (2021); TDR
Any opioid present	Opiates present (Council) / any opioid present (EUDA)	2012–2017 / 2021, 2022, 2024	NR2014, EMCDDA (2019) / EUDA tables
Single-substance deaths	Council attribution of death to one substance	2017–2024 (2015 partial)	TDR (EDR 2017)
Polysubstance deaths	Council attribution to more than one substance, incl. drug–alcohol	2016–2024	TDR
Population	Year-end resident population, all ages and 15–64	2010–2024	TurkStat ADNKS
European reference values and country tables	EU totals, rates, male share, opioid involvement, age distribution	2021–2024	EUDA source tables, EDR 2023–2026; FAQ 2024–2025

TDR, Turkish Drug Report; NR2014, 2014 Reitox national report (2013 data); CDR, EMCDDA Country Drug Report; EDR, European Drug Report; ADNKS, Address Based Population Registration System.

Supplementary Table S2

Population denominators, Türkiye, 2010–2024.

Year	Population, all ages	Population, ages 15–64
2010	73,722,988	48,618,564
2011	74,724,269	49,516,670
2012	75,627,384	50,346,979
2013	76,667,864	51,088,202
2014	77,695,904	51,926,356
2015	78,741,053	52,640,512
2016	79,814,871	53,359,594
2017	80,810,525	54,881,652
2018	82,003,882	55,633,349
2019	83,154,997	56,391,925
2020	83,614,362	56,592,570
2021	84,680,273	57,459,186
2022	85,279,553	58,092,773
2023	85,372,377	58,337,938
2024	85,664,944	58,626,081

All-age populations (TurkStat) are the denominators of the all-age rates in Table 1. Populations aged 15–64 for 2010–2016 are Eurostat denominators, as used in the European comparison, and for 2017–2024 TurkStat denominators. Rates at ages 15–64 are given in Supplementary Table S3.

Supplementary Table S3

European comparator panel: composition, pooled mortality rates and Türkiye-to-panel rate ratios, 2010–2022.

Year	Panel deaths	Panel population 15–64	Panel rate /10⁶	Türkiye rate /10⁶	Crude ratio	Sex- and age-standardised ratio	Panel males 15–24 /10⁶	Türkiye males 15–24 /10⁶	Countries with complete data, n	Ratio, all complete countries
2010	1290	72,872,717	17.7	2.6	0.14	0.14	23.9	3.3	22	0.17
2011	1342	73,021,140	18.4	2.0	0.11	0.10	26.2	3.4	23	0.13
2012	1427	72,872,254	19.6	3.1	0.16	0.13	23.3	7.2	23	0.20
2013	1393	72,763,147	19.1	4.4	0.23	0.20	21.8	10.4	22	0.29
2014	1488	72,580,278	20.5	9.2	0.45	0.39	26.0	24.2	22	0.58
2015	1639	72,284,510	22.7	10.1	0.45	0.39	24.4	24.3	23	0.60
2016	1586	72,036,919	22.0	14.6	0.66	0.58	21.4	36.8	22	0.90
2017	1678	72,192,237	23.2	16.7	0.72	0.64	22.1	35.5	22	0.85
2018	1706	72,076,615	23.7	11.6	0.49	0.44	23.6	22.1	22	0.60
2019	1745	71,990,673	24.2	6.0	0.25	0.22	24.9	16.9	23	0.32
2020	1701	71,780,467	23.7	5.5	0.23	0.21	30.3	9.2	24	0.29
2021	1678	71,483,539	23.5	4.8	0.20	0.18	27.9	6.8	22	0.24
2022	1796	71,366,695	25.2	4.2	0.17	0.15	26.4	5.6	16	0.17

Fixed panel: Austria, Croatia, Cyprus, Finland, Italy, Lithuania, Luxembourg, the Netherlands, Slovenia and Sweden, the EU countries with sex- and age-specific counts reported for both sexes and all three adult age bands (15–24, 25–44, 45–64) in every year 2010–2022. Sex- and age-standardised ratio: sex- and age-specific rates for Türkiye applied to the panel population structure of the same year, divided by the panel rate. The last two columns pool all EU countries with complete data in the given year (France to 2016, Poland to 2021, Germany and Spain from 2021, Italy throughout). Counts for Türkiye in 2014–2017 exclude cases with unrecorded age or sex (18, 57, 141 and 34 cases), so the rates and ratios for those years are slightly understated. Source: EUDA (2024), Frequently asked questions: drug-induced deaths in Europe, source tables (deaths reported under the drug-related deaths protocol; Eurostat denominators). The 2011–2012 values in the European tables equal those in the 2014 Reitox national report, which names the CFM as source; 2010 is reported through the same single channel. In the European Drug Report 2026 narrative, synthetic cannabinoid deaths for 2024 are given as 202 against 204 in the national report, a two-case channel difference.

Supplementary Figure S1

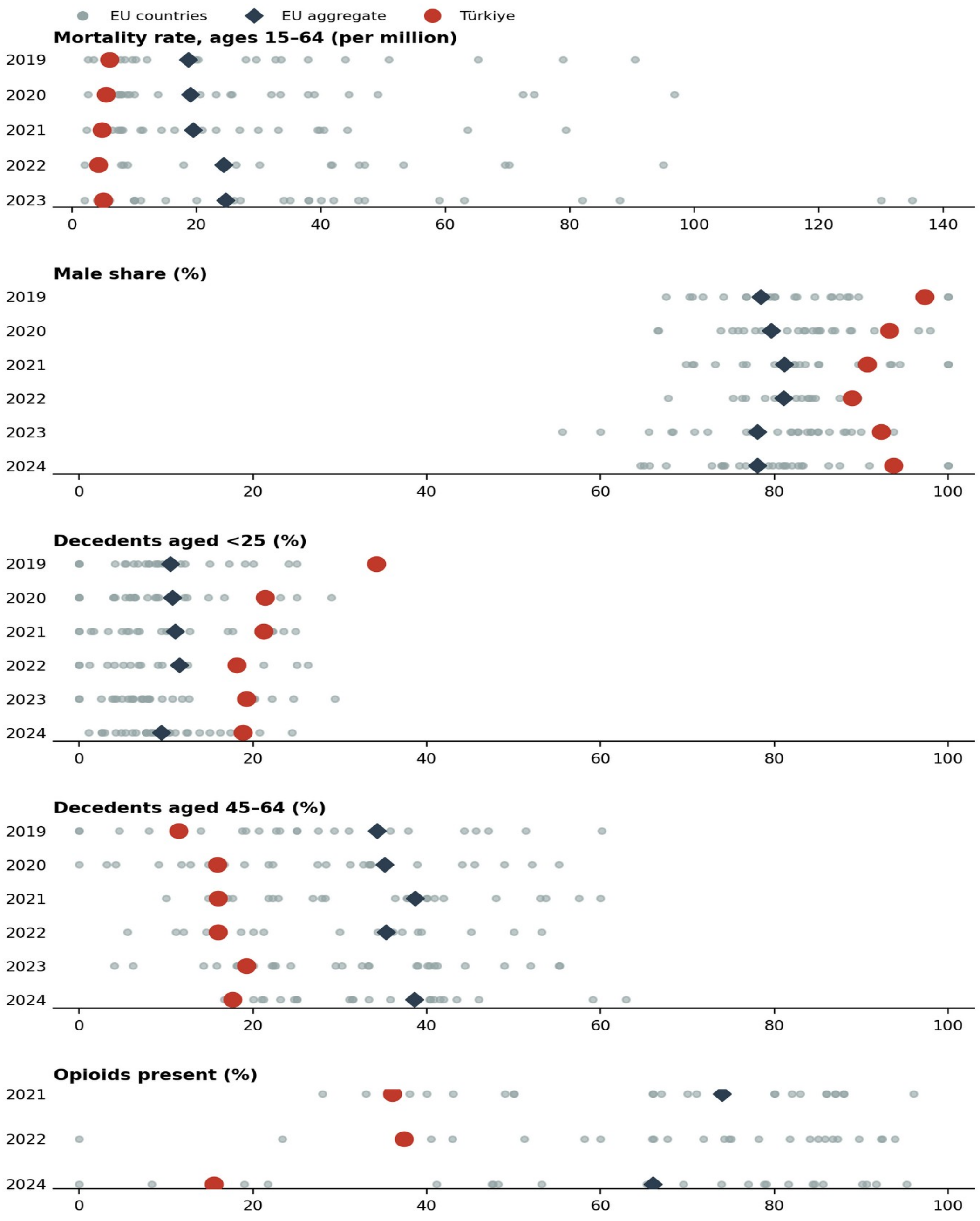

Türkiye against the European distribution, 2019–2024. Each row shows one year; grey dots are individual EU countries, diamonds the EU aggregate and red dots Türkiye. Mortality rate, male share and age distribution for 2019–2022 are computed from the sex- and age-specific counts reported to the EUDA for persons aged 15–64 (Eurostat denominators; aggregate pooled over the countries shown; age shares are shares of deaths at ages 15–64). 2023 values are from the EUDA (2025b) tables (EU rate 24.7 per million; rates rounded by the source) and 2024 values from the European Drug Report 2026 tables (EUDA, 2026) (age shares of all deaths). Opioid involvement (any opioid, as reported to the EUDA) is shown for 2021, 2022 and 2024; no EU aggregate was published with the 2022 country table.

Supplementary Methods

Analytical coverage. In 2013, synthetic cannabinoid analysis was unavailable in six of the CFM's ten laboratories. Istanbul, Trabzon and Izmir could analyse JWH-018 and JWH-073, while Ankara additionally covered JWH-122 and related metabolites (Reitox National Focal Point Türkiye, 2014). This establishes substantial geographic and analyte limitations at the start of the series. Published Turkish studies in other settings illustrate related analytical constraints. Gurdal et al. (2013) analysed seized herbal products, Bozkurt et al. (2014) examined clinical urine samples with an enzyme immunoassay, and Atasoy et al. (2021) reanalysed routine immunoassay-negative probation urine samples using targeted LC–MS/MS. These studies do not establish the year-by-year chronology of CFM post-mortem testing. The market subsequently shifted to successive generations of analogues, including the AB-/ADB-FUBINACA and MDMB families notified through the EU Early Warning System (EMCDDA, 2021). Because targeted detection depends on panel inclusion and the availability of certified reference standards, newly circulating compounds may have remained undetected until methods were updated. No systematic record of annual CFM panel coverage, instrumentation or detection limits has been published.

S-M1. Consistency of the data across publication channels

Annual totals for 2010–2024 were identical in the Turkish Drug Reports (2018–2025 editions), in the 2014 Reitox national report (2007–2013) and in the EUDA trend tables (2010–2024) for every overlapping year. The sex- and age-specific counts reported to the EUDA for persons aged 15–64 summed to 637, 333, 309 and 269 for 2018–2021, identical to the national counts, and to 243 for 2022 against a national count of 244. For 2014–2017 the sex- and age-specific counts summed to 479, 533, 779 and 907 against national totals of 497, 590, 920 and 941; the shortfalls (18, 57, 141 and 34) correspond to cases with unrecorded age or sex and are the reason the EMCDDA-published rates for 2015 (10.1 per million) and 2016 (14.6 per million) are lower than the rates computed from national totals. The 2014 Reitox national report reports 8 deaths outside the 15–64 age range or with unknown age in each of 2011, 2012 and 2013, exactly matching the difference between the EUDA sex- and age-specific sums and the national totals for those years.

S-M2. Values obtained from a published chart

Figure 11 of the EMCDDA expert-network update (EMCDDA, 2019a) reproduces the Council of Forensic Medicine (CFM) annual counts of deaths with opioids, deaths without opioids and deaths with synthetic cannabinoids for 2008–2017 as a line chart with gridlines at intervals of 100 deaths. The page was rasterised at 300 dots per inch; the nine horizontal gridlines (0–800) were located as rows of light grey pixels spanning the plot area; the year positions were taken from the axis labels; and for each series and year the median vertical position of the coloured line pixels within ±5 pixels of the year position was converted to deaths by linear interpolation between gridlines. Validation against published counts: synthetic cannabinoids 2012 (chart 7, published 7), 2013 (36, 35), 2015 (145, 137), 2016 (373, 377), 2017 (551, 564); opioids 2013 (164, 164, Reitox national report); sums of the opioid and non-opioid series 2008–2017 within 7 deaths of the national totals in every year. The maximum deviation was 13 deaths (2017). Values used in the manuscript from this source: synthetic cannabinoids 2014 (99); opioids 2014 (286), 2015 (315), 2016 (292) and 2017 (219).

S-M3. Change-point selection and fixed-knot sensitivity analysis

All configurations of zero to three change points at years 2012–2022 with at least two years between change points (141 configurations) were fitted as Poisson regressions of annual deaths on calendar year with a log-population offset. The configuration with change points at 2017, 2019 and 2022 had the lowest Bayesian information criterion (237.6; Pearson dispersion 10.9). The selected configuration was compared with the nearest alternatives. ΔBIC values relative to the selected model were: (2015, 2017, 2020) +12.3; (2015, 2017, 2021) +12.9; (2017, 2019, 2021) +15.4; (2012, 2016, 2022) +16.6; best two-change-point model (2016, 2022) +33.9. A ΔBIC above 10 is conventionally regarded as very strong evidence; the selected configuration is therefore not a marginal choice. Six of the ten best configurations include 2017,

and the near-optimal configurations place the peak between 2015 and 2017. Under the best two-change-point model the series still shows growth to a mid-decade peak (+49.8% per year to 2016), decline (−23.7% per year to 2022) and renewed growth (+42.4% per year), so the qualitative narrative does not depend on the number of change points. Confidence intervals for change-point locations cannot be reliably estimated from fifteen annual observations; the range of near-optimal configurations above serves as the practical expression of that uncertainty, and inference is conditional on the selected configuration. A model with conventional fixed change points at 2017 and 2022 (BIC 341.5; dispersion 19.5) gave annual percent changes of +35.3% (95% CI +25.0 to +46.4) for 2010–2017, −30.3% (−37.0 to −22.8) for 2017–2022 and +57.9% (+15.1 to +116.5; p = .009) for 2022–2024. The qualitative pattern (steep rise, decline, renewed rise) is the same; the selected model additionally separates the 2017–2019 collapse from the 2019–2022 plateau, which the fixed-knot model averages into a single decline. For the ratio of the Turkish rate to the panel rate, the segmented model gave +33.8% per year for 2010–2017 (95% CI +26.0 to +42.1), −43.3% per year for 2017–2019 (−48.6 to −37.5) and −12.9% per year for 2019–2022 (−15.6 to −10.1).

S-M4. Sensitivity to cases with unrecorded age or sex.

The sex- and age-specific counts reported to the EUDA for 2014–2017 exclude cases whose age or sex was not recorded: 18, 57, 141 and 34 cases respectively. Extreme-assignment bounds were computed by assigning all unrecorded cases first to one category and then to the other. For the female share, the recorded values of 6.3%, 6.6%, 4.4% and 3.4% have bounds of 6.0–9.7%, 5.9–15.6%, 3.7–19.0% and 3.3–6.9%; even under the implausible extreme in which every unrecorded case is female, the male share remains at least 81% in every year. For the share of decedents under 25, the recorded values of 35.7%, 32.6%, 32.3% and 27.6% have bounds of 34.4–38.0%, 29.5–39.2%, 27.4–42.7% and 26.6–30.2%. The conclusions that the wave was overwhelmingly male and concentrated at young ages hold at either extreme. Trend statistics for sex and age use recorded cases only, and the sex- and age-specific rates for 2014–2017 are accordingly slight underestimates.

References cited in this supplement

Atasoy, A., Daglioglu, N., Gören, İ. E., Girisbay, A., Aslan, R., & Akgur, S. A. (2021). Determination of synthetic cannabinoids in randomly urine samples collected from probationers in Turkey. Forensic Science International, 322, 110752. https://doi.org/10.1016/j.forsciint.2021.110752

Bozkurt, M., Umut, G., Evren, C., & Karabulut, V. (2014). Clinical characteristics and laboratory test results of patients admitted to outpatient clinic for synthetic cannabinoid usage. Düşünen Adam: The Journal of Psychiatry and Neurological Sciences, 27, 328–334. https://doi.org/10.5350/DAJPN2014270407

Emniyet Genel Müdürlüğü Narkotik Suçlarla Mücadele Başkanlığı [EGM]. (2025). 2025 Türkiye uyuşturucu raporu [2025 Turkish drug report]. Turkish National Police. (And earlier editions, 2018–2024, for the corresponding data years.)

European Monitoring Centre for Drugs and Drug Addiction. (2019a). Drug-related deaths and mortality in Europe: Update from the EMCDDA expert network. Publications Office of the European Union. https://doi.org/10.2810/627691

European Monitoring Centre for Drugs and Drug Addiction. (2021). Synthetic cannabinoids in Europe: A review. Publications Office of the European Union.

European Union Drugs Agency. (2024). Drug-induced deaths in Europe: Frequently asked questions [Data tables].

European Union Drugs Agency. (2025b). Drug-induced deaths in Europe: Frequently asked questions [Data tables].

European Union Drugs Agency. (2026). European drug report 2026: Trends and developments. Publications Office of the European Union.

Gurdal, F., Asirdizer, M., Aker, R. G., Korkut, S., Gocer, Y., Kucukibrahimoglu, E. E., & Ince, C. H. (2013). Review of detection frequency and type of synthetic cannabinoids in herbal compounds analyzed by Istanbul Narcotic Department of the Council of Forensic Medicine, Turkey. Journal of Forensic and Legal Medicine, 20, 667–672. https://doi.org/10.1016/j.jflm.2013.03.041

Reitox National Focal Point Türkiye. (2014). 2014 national report (2013 data) to the EMCDDA: Turkey. Turkish National Police Counter Narcotics Department / TUBİM.

Turkish Statistical Institute [TurkStat]. (2025). Adrese dayalı nüfus kayıt sistemi sonuçları, 2024 [Address based population registration system results, 2024]. TurkStat.
